# Professorial Changes

**Published:** 1867-12

**Authors:** 


### *Professorial Changes.*

We congratulate the profession on the recognition of the highest order of merit, in the neighborhood of New York City.

SAMUEL G. ARMOR, M.D., late of the Medical Department of the University of Michigan, has been appointed to the Chair of Principles and Practice of Medicine in the Long Island College, at Brooklyn, Prof. Flint having resigned the position in order to pay more especial attention to Clinical Medicine.

CORYDON L. FORD, M.D., late of Michigan, also has been appointed to and has accepted the Chair of Anatomy, &c., in the same College.

Two better selections for any school could not have been made. Each of these gentlemen is of that sort referred to by the old writer: *Non tetigit quod non ornavit.*

We had "indulged a trembling hope" of standing side by side with this *par nobile fratrum* in the future, and we greet their exit to the East with the reflection that our loss is its gain—much needed.

